# Hand, Foot, and Mouth Disease Outbreak and Coxsackievirus A6, Northern Spain, 2011

**DOI:** 10.3201/eid1904.121589

**Published:** 2013-04

**Authors:** Milagrosa Montes, Juncal Artieda, Luis D. Piñeiro, Marina Gastesi, Inmaculada Diez-Nieves, Gustavo Cilla

**Affiliations:** Author affiliations: Hospital Universitario Donostia-Instituto Biodonostia, San Sebastián, Spain (M. Montes, L.D. Piñeiro, G. Cilla);; Biomedical Research Centre Network for Respiratory Diseases, San Sebastián (M. Montes, G. Cilla);; Public Health Division of Gipuzkoa, Basque Government, San Sebastián (J. Artieda);; Research Centre Network for Epidemiology and Public Health, San Sebastián (J. Artieda);; Health Centre of Osakidetza Basque Health Service, Irun, Spain (M. Gastesi, I. Diez-Nieves)

**Keywords:** Hand foot and mouth disease, outbreak, coxackievirus, infections, molecular epidemiology, Spain, viruses

**To the Editor:** Hand, foot, and mouth disease (HFMD) is an acute, febrile viral infection characterized by vesicular exanthema on the palms of the hands, soles of the feet, and oral mucosa. The infection is transmitted through oral and respiratory secretions, vesicular fluid, and/or feces of affected persons. The most common etiologic agents are coxsackievirus (CV) A16 and human enterovirus (HEV) 71, but other HEVs, mainly belonging to species A, have also been associated with illness (1). HFMD mainly affects infants and children <5 years of age.

On May 10, 2011, an outbreak of HFMD was reported in a daycare center in the city of Irun in Basque Country, Spain. Monitoring subsequently was conducted for HFMD cases among children in the health district that contained the daycare center (a total of 4,540 children <14 years of age). Children with fever and vesicular rash on the palms and/or soles and in the mouth were considered HFMD patients. Pharyngeal and/or dermal exudate and/or feces were collected for virologic confirmation from 37 representative HFMD patients (17 with multiple specimens) selected by sentinel pediatricians in outpatient clinics. Viral RNA was extracted directly from specimens (NucliSENS Easy-Mag, Bio-Mèrieux, Marcy-l’Étoile, France) and was used in the amplification methods. Enterovirus RNA was detected by an in-house real-time PCR that amplified a fragment within the 5′ untranslated region by using described primers (2). For genotyping, the viral protein 1 gene was amplified by using described methods (3), followed by partial sequencing of the obtained amplicons by using the 3130XL Genetic Analyzer (Applied Biosystems, Foster City, CA, USA). Control measures recommended were frequent and careful handwashing with soap and running water by children and staff and increasing the cleaning of surfaces and objects in daycare centers and nursery schools.

During April–September 2011, a total of 99 cases of HFMD were notified; 53 patients were boys. Twenty-five cases occurred in the daycare center, all before May 13 (attack rate 55.6%), and 74 were community acquired, occurring mainly after that date. All cases occurred in children <4 years of age (median age 1.8 years; incidence 77 cases/1,000 inhabitants). The highest incidence occurred in children 12–36 months of age (122.4 cases/1,000 inhabitants). In addition to a papulovesicular rash on the palms, soles, and/or buttocks, 89 (90%) HFMD patients showed a perioral papulovesicular rash that did not extend to the rest of the face. None of the children were hospitalized. 

Enterovirus was detected in 49 samples (28 pharyngeal, 2 dermal, 19 fecal) from 33 HFMD patients. For 30 of these patients, the samples were sufficient for genotyping. CVA6 was detected in 27 (90%) patients and CVA10 in 2 (7%) patients; for 1 patient, no genotype was obtained. Seven (7%) of the 99 children with HFMD were brought for medical assistance for onychomadesis during the 9–67 days after the HFMD episode. In 2 of them, HFMD had been virologically confirmed as being caused by CVA6.

Our results suggest that CVA6 can cause HFMD outbreaks that develop rapidly and reach a high incidence in children. Despite the mildness of the disease, the high attack rate in the daycare center alarmed families and staff. HFMD is not subject to epidemiologic surveillance in Spain, and thus its real incidence cannot be identified.

Although CVA6 has long been known to cause HFMD (1), it has not usually been considered to play a major role in this disease. Except in a few countries, CVA6 has been infrequently detected until recent years. However, since 2008, this virus has caused major outbreaks of HFMD in some countries of eastern Asia and Europe and, more recently, in the United States (4–9); the CVA6 strains in this outbreak shared >97% of nucleotide identities in the viral protein 1 gene and showed sequence similarity >94% with the strains that caused these outbreaks. These strains segregated in a phylogenetic tree (Figure), supporting the recent international spread of emerging CVA6 genetic variants (4). In Taiwan and Japan, the emergence of these strains has been associated with a change in the predominant clinical expression of the infections produced by CVA6, from herpangina before 2009 to HFMD in 2010–2011 (7,8). The development of a perioral rash has also been associated to HFMD caused by CVA6 (10). 

**Figure:**
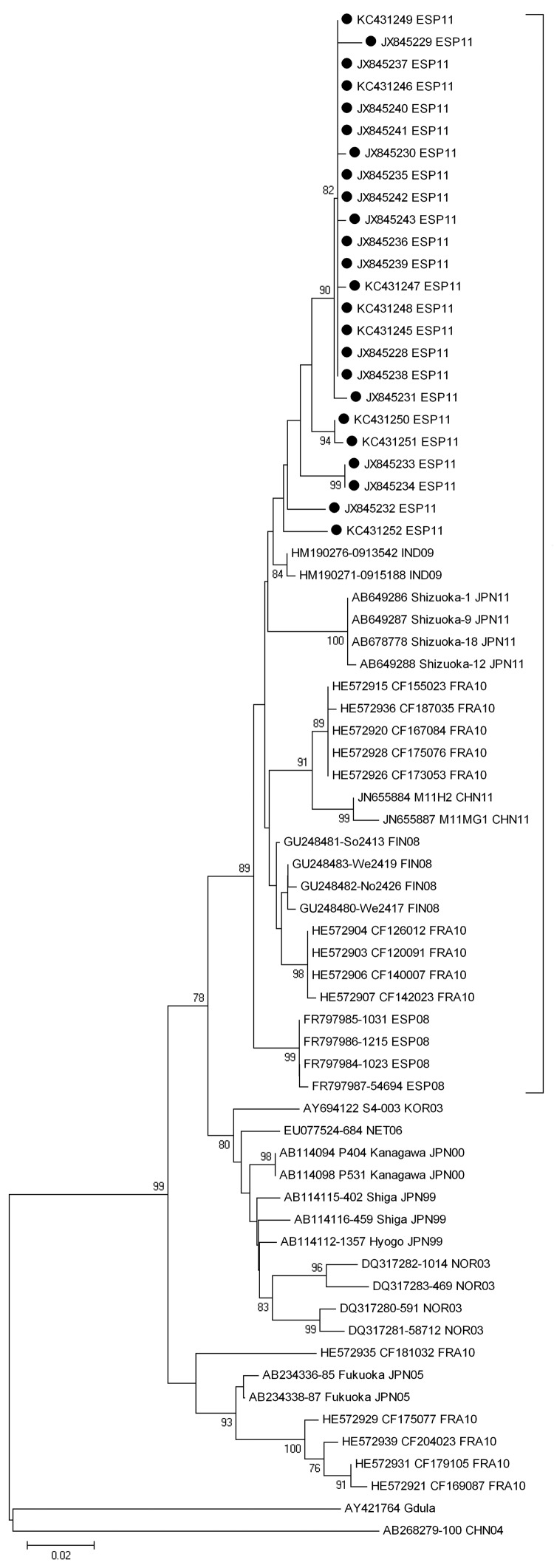
Phylogenetic analysis of the partial viral protein 1 gene sequence (positions 2929–3348, based on strain Shizuoka-18, GenBank accession no. AB678778) of coxsackievirus A6 isolated from distinct patients with hand, foot, and mouth disease detected in Irun, Spain, April–September 2011, compared with the Gdula prototype strain and other representative strains. Black dots indicate the strains in this study (GenBank accession nos. JX845228–JX845243 and KC431245–431253). The tree was constructed by using the neighbor-joining method with 1,000 bootstrap replications and shows bootstrap values >75%. Genetic distances are based on pairwise analysis by using the Kimura 2-parameter method in MEGA5.1 software (www.megasoftware.net). Bracket indicates strains showing nucleotide identity >94% and detected in outbreaks during 2008–2011. Scale bar indicates the number of substitutions per nucleotide position.

Although the course of HFMD is usually self-limiting, illness and death rates vary among outbreaks. Severe illness is more frequent in outbreaks caused by HEV71 (1); in outbreaks caused by CVA6 in Taiwan and the United States, the illness affected a broader spectrum of skin sites and was associated with more severe and extensive rash than was HFMD caused by other coxsackieviruses (7,9).

In conclusion, reports of HFMD outbreaks associated with CVA6 are increasing. Improved HFMD surveillance is required, with virus genotyping as a key element.
